# Impf-Guides – experiences with a project aimed at increasing vaccination willingness in Munich during the COVID-19 pandemic

**DOI:** 10.3205/zma001839

**Published:** 2026-04-15

**Authors:** Jan M. Zottmann, Johanna Huber, Matthias J. Witti, Fabian Jacobs, Tobias Mahl, Alexander Schmidt, Martin R. Fischer

**Affiliations:** 1 LMU University Hospital, LMU Munich, Institute of Medical Education, Munich, Germany; 2 District Court Munich II, Munich, Germany; 3 LMU Munich, Student Council of Human Medicine, Munich, Germany

**Keywords:** vaccination willingness, health communication, health literacy, evaluation, COVID-19

## Abstract

**Objective:**

The project *Impf-Guides* aimed at motivating citizens in selected districts of Munich to get vaccinated against COVID-19 by providing information and advice, thereby increasing the vaccination rate among the population.

**Project description:**

The project began in February and ended in December 2022. A total of 31 medical students from LMU Munich were recruited and trained as *Impf-Guides*. This training included communication and de-escalation techniques. The students conducted consultations, promoted mobile vaccination campaigns and provided on-site support in city districts with defined social challenges. For quality assurance purposes, accompanying evaluations of the vaccinated individuals and the *Impf-Guides* were carried out.

**Results:**

As part of the mobile vaccination campaigns, 351 people were vaccinated, 18% of whom took part in the evaluation. The majority received their third or fourth vaccination and were satisfied with the consulting activities provided by the *Impf-Guides.* Of the 31 *Impf-Guides*, only eight took part in the evaluation. Few students experienced challenging situations (e.g. language barriers) or conflicts (e.g. vaccine hesitancy) during their consultancy work. The students considered the project useful with regard to their future medical practice.

**Discussion and conclusion:**

The project mainly reached people who were already willing to be vaccinated. The aim of the project to significantly increase vaccination willingness was ultimately not achieved. The extent to which the *Impf-Guides* contributed to the health literacy of the population remains speculative. Regardless of vaccination campaigns, consideration should be given to whether trained students could advise the population on general health, prevention and health promotion issues in the future.

## Zusammenfassung

**Zielsetzung:**

Ziel des Projekts *Impf-Guides *war es, Bürger:innen ausgewählter Stadtteile Münchens durch Information und Beratung zur COVID-19-Impfung zu motivieren und so die Impfquote in der Bevölkerung zu erhöhen.

**Projektbeschreibung:**

Das Projekt begann im Februar und endete im Dezember 2022. Es wurden 31 Medizinstudierende der LMU München als *Impf-Guides *rekrutiert und geschult. Die Schulung beinhaltete auch ein Training zu Kommunikations- und Deeskalationstechniken. Die Studierenden arbeiteten in Stadtteilen mit definierten sozialen Herausforderungen. Dort führten sie Beratungsgespräche, bewarben mobile Impf-Aktionen und unterstützten diese vor Ort. Zur Qualitätssicherung wurden begleitende Evaluationen der Geimpften sowie der *Impf-Guides *durchgeführt.

**Ergebnisse:**

Im Rahmen der mobilen Impf-Aktionen wurden 351 Personen geimpft, von denen 18% an der Evaluation teilnahmen. Die Mehrheit erhielt die dritte oder vierte Impfung und war mit der Beratungstätigkeit der *Impf-Guides* zufrieden. Von den 31 *Impf-Guides *nahmen acht an der Evaluation teil. Nur wenige Studierende erlebten bei ihrer Beratungstätigkeit herausfordernde (z. B. Sprachbarrieren) oder konfliktreiche Situationen (z. B. Impfskepsis). Im Hinblick auf ihre spätere ärztliche Tätigkeit schätzten die Studierenden das Projekt als nützlich ein.

**Diskussion und Schlussfolgerung:**

Das Projekt erreichte überwiegend Personen, die bereits impfwillig waren. Das Projektziel, die Impfbereitschaft deutlich zu erhöhen, konnte nicht erreicht werden. Inwieweit das Projekt zur Gesundheitskompetenz der Bevölkerung beigetragen hat, bleibt spekulativ. Unabhängig von Impfkampagnen sollte überlegt werden, ob geschulte Studierende die Bevölkerung künftig zu allgemeinen Gesundheits-, Präventions- und Gesundheitsförderungsthemen beraten könnten.

## 1. Objective

The *Impf-Guides *project was launched in early February 2022 on the initiative of the Dean of Studies in Human Medicine at LMU Munich and the City of Munich Health Department, in cooperation with the Student Council of Human Medicine at LMU Munich, amid considerable media interest [1], [2], [3]. The project name combines the German verb *impfen* (“to vaccinate”) with the English word *guides*. The aim of this project was to increase the willingness of Munich citizens to be vaccinated by providing targeted information, consultation and vaccination services, thereby helping to increase the comparatively low rate of first and second vaccinations among the population of Munich against the coronavirus disease (COVID-19) caused by the SARS-CoV-2 virus [4], [5], [6]. At the beginning of 2022, the rate for first vaccinations was 71.5%, for second vaccinations 68.4%, and 38% for booster vaccinations in Munich [1]. Similar campaigns were carried out with great success in other federal states and cities in Germany and served as a blueprint for the project [7].

The 5C model developed by Betsch and colleagues identifies five psychological reasons that significantly influence vaccination decisions (trust, risk perception, barriers to implementation, extent of information seeking, sense of responsibility for the community) [8], [9]. Accordingly, the project was based on the assumption that the insufficient willingness to be vaccinated among the Munich population could be attributed, on the one hand, to a lack of information about vaccination and, on the other hand, to a lack of access to the health system, for example due to socioeconomic factors [9], [10]. Medical students from LMU Munich were therefore tasked with acting as *Impf-Guides,* visiting city residents at selected locations to provide them with information and advice on COVID-19 vaccination. The focus was on the practical benefits of increasing vaccination willingness among the population of Munich rather than on obtaining scientific findings. It was explicitly not the aim of the project to convince vaccine sceptics of the necessity of vaccination. 

## 2. Project description

### 2.1. Recruitment and training of the “Impf-Guides”

The City of Munich provided funding for the remuneration of up to 50 *Impf-Guides* for the project period from the beginning of February to the end of December 2022. Over the course of the project, a total of 31 medical students were recruited, who received student assistant contracts for a maximum of 19 hours per week for their work. Recruitment took place among students in the clinical study phase at the LMU and Technical University (TU) Munich campuses and was the responsibility of the Student Council of Human Medicine at LMU Munich. The council was supported by staff from the Institute of Medical Education (DAM) at LMU University Hospital. The project was advertised from February to May 2022 through word of mouth, at semester introductions and on MeCuMplus [11], an information portal for medical students at LMU. In addition, the student council organised four onboarding events in cooperation with the City of Munich Health Department and the DAM. The aim of these events was to inform interested students about the project and then recruit them as *Impf-Guides*. The deployment was coordinated by a DAM employee in consultation with the city’s project staff.

In preparation for their work in the field, the future *Impf-Guides* had to complete a one-day training course. The training was conceptualised and organised jointly by employees of the City of Munich and the DAM. Information about the training was provided in advance on the Med.Moodle learning platform and continuously updated. The face-to-face training was held twice and attended by a total of 22 *Impf-Guides*. The following topics were covered in the training:

1. Introduction to the project and information about city districts
2. Lecture on offering conversations in public spaces
3. Lectures on “vaccination myths” and the implementation of COVID-19 vaccination in Munich
4. Communication and de-escalation training (a more detailed overview of the training programme, the course content and the lecturers can be found in attachment 1 )

The theoretical parts of the lectures were recorded so that the students who did not want to or were unable to work as *Impf-Guides* right from the start of the project could prepare for their work with the help of the online materials. In addition, *Impf-Guides *who had already been trained could access these online materials at any time to refresh their knowledge. After the *Impf-Guides* had completed their first assignments in the field, they were able to participate in two voluntary supervision sessions. These sessions were conducted by the lecturer of the communication and de-escalation training (see attachment 1 ). The supervision sessions provided an opportunity to discuss challenging situations, problems and questions. For medical questions, the *Impf-Guides* could contact the medical service directors of the Munich vaccination centres. 

### 2.2. Consulting and vaccinating citizens

The city of Munich staff selected the locations for the *Impf-Guides* based on social challenges in the city districts (including the proportion of recipients of social welfare and the proportion of people with a migrant background) and the infection rates there [10], [12]. City officials assumed that citizens in these disadvantaged neighbourhoods have limited access to the healthcare system and therefore refuse vaccination due to ignorance and/or fear [9].

In preparation for the work of the *Impf-Guides*, citizens in the selected city districts were informed about the project and the planned mobile vaccination campaigns by means of multilingual mailings from the city of Munich (a total of more than 175,000 mailings were sent out in German, English, French, Croatian, Russian and Turkish language). One week before their assignment there, the *Impf-Guides* visited a district to identify suitable consultation locations such as public places or social institutions in the district. Consultations usually took place in the first half of the week – preferably in mixed-gender tandems to increase acceptance. The mobile vaccination campaigns, which the *Impf-Guides* referred to during their conversations, followed in the second half of the week. The medical staff of the Aicher Group (an emergency medical service commissioned by the city for the Munich metropolitan area), who ran the vaccination centres in Munich in cooperation with emergency medical services provider MKT, carried out the mobile vaccination campaigns [9]. The *Impf-Guides *supported these campaigns on site by helping citizens willing to be vaccinated to fill out the paperwork (information and medical history forms) and offering a free health check (blood pressure and blood sugar measurement, weighing) after the vaccination. These health checks served as an additional incentive in the consultancy sessions to make the vaccination offer more attractive. 

### 2.3. Evaluation tools

As part of the project, two accompanying online surveys were conducted for quality assurance purposes and to evaluate the measures implemented. Due to time constraints, it was not possible to pilot the evaluation tools that had been developed. A survey of vaccinated citizens on site was used to evaluate the consulting activities of the *Impf-Guides* and the mobile vaccination campaigns. The second survey was aimed at the *Impf-Guides *themselves and sought to capture the students' experiences in the field. Participation in the surveys was voluntary and anonymous. Both surveys were administered using the web-based evaluation software evasys, version 8.2. For the presentation of results, frequencies (absolute and relative) were calculated for single and multiple choice questions, and mean values and standard deviations were calculated for Likert scale questions. Free text responses were summarised in terms of content. All data was stored in a password-protected web-based cloud storage system at the Leibniz Supercomputing Centre. Access was restricted to project staff. The surveys of vaccinated individuals and students were approved in advance by the Ethics Committee of LMU Munich (application number 22-0333). 

#### 2.3.1. Evaluation by vaccinated citizens 

To assess the success of the advice provided by the *Impf-Guides *and the subsequent mobile vaccination campaigns, an online evaluation of vaccinated individuals was conducted. With regard to the survey of vaccinated individuals, the evaluation was deliberately not conducted immediately after the sensitive consultancy sessions, as this might have reinforced reservations observed in previous surveys (e.g. that the pandemic serves as a pretext for the state and its institutions to collect personal data [13]) and thus could have counteracted the consultancy sessions.

The main questions in the survey of vaccinated individuals were: Did the *Impf-Guides *contribute to the decision to get vaccinated? Why did the citizens ultimately decide to get vaccinated? How can the group of vaccinated individuals be described? To this end, the authors developed a questionnaire based on existing surveys on factors that promote and hinder willingness to be vaccinated [13], [14]. It contained 36 questions, consisting of single-choice, multiple-choice and free text questions as well as Likert-scaled statements (“do not agree at all”=1 to “fully agree”=5). The complete questionnaire in English is attached as attachment 2 . It was available in six languages, corresponding to the city’s mailings. Vaccinated individuals could participate in the survey using a QR code either on their own mobile devices or on a tablet provided by the DAM Institute. In addition, the *Impf-Guides* were available as contact persons for questions and problems during the completion of the evaluation. The questionnaire was designed so that it could be completed within the waiting period after vaccination (approx. 10 minutes). 

#### 2.3.2. Evaluation by the “Impf-Guides” 

The experiences of the students were examined as part of the second semi-qualitative online evaluation of the *Impf-Guides.* The main questions were: What challenges did the *Impf-Guides *face when advising citizens on the street? To what extent was the training helpful in overcoming these challenges? What content was still missing from the training? How do the *Impf-Guides *subjectively assess the success of their work? As these questions are specifically tailored to the project described here, the authors were unable to draw on tried-and-tested instruments for this evaluation. The questionnaire for the students is attached as attachment 3 and consisted of 38 questions, 15 of which are free-text questions and 23 are single-choice questions and Likert-scale statements (“completely applies”=1 to “do not apply at all”=5). At the end of the project, the students were asked by email invitation from the Dean of Studies in Human Medicine at LMU Munich and the project management to take 20 to 30 minutes to complete the evaluation. The students were reminded several times to participate in the evaluation. 

## 3. Results

The project began in February and ended in December 2022. In total, the *Impf-Guides* visited 12 districts of Munich. In July, the last mobile vaccination campaign took place due to declining demand among the Munich population. Although the *Impf-Guides* continued to provide advice after the last mobile vaccination campaign, citizens willing to be vaccinated then had to visit the city's vaccination centres. As part of the mobile vaccination campaigns, 351 people had received a vaccination by July 2022. 

### 3.1. Results of the evaluation by vaccinated citizens

Of the 351 people who were vaccinated as part of the mobile vaccination campaigns, 63 vaccinated citizens completed the questionnaire to evaluate the project (corresponding to a response rate of approximately 18%). It is not possible to estimate how many people were informed about the mobile vaccination campaigns but eventually took advantage of an alternative vaccination offer (e.g. at one of the vaccination centres or at their general practitioner). As no more mobile vaccination campaigns were carried out after July 2022, it was no longer possible to survey vaccinated citizens from that point onwards. Of those vaccinated, 63.5% were over 40 years of age and almost as many spoke German as their native language (60.3%). Table 1 (Tab. 1) summarises the sociodemographic data of the vaccinated individuals. 

The majority of those vaccinated received their third (41.3%) or fourth (41.3%) COVID-19 vaccination. Only 7.9% were vaccinated for the first time and 3.2% for the second time. The statement “the vaccination offer in my district made the decision to vaccinate easy” (5-point Likert scale; 1 “do not agree at all”, 5 “fully agree”) was rated by 51 respondents with an average of 4.8 (*SD*=0.6). Almost half of those vaccinated (42.9%) had become aware of the vaccination campaign through the city's mailings. Other sources of information were the *Impf-Guides *(17.5%), neighbours (11.1%) and family/friends (7.9%). Almost half of the respondents (47.6%) received a consultation before being vaccinated; however, more than half (56.7%) said they would have been vaccinated even without a consultation. The work of the *Impf-Guides* was rated positively by the majority (see table 2 (Tab. 2)). The questionnaire concluded with free text question asking why the participants had only now decided to get vaccinated. Of the 34 free text responses collected, the most common theme was “protecting oneself from illness or severe symptoms and protecting others” (22 mentions).

Another set of questions asked about the respondents' possible concerns and impressions regarding the COVID-19 pandemic, e.g. with regard to their health and financial situation or their confidence in science to solve the COVID-19 crisis, see table 3 (Tab. 3).

### 3.2. Results of the evaluation by the “Impf-Guides”

From the end of May 2022 to the end of July 2022, eight of the 31 *Impf-Guides* took part in the evaluation of their work. The first set of questions concerned the face-to-face training and the supervision offered. Of the eight *Impf-Guides*, six took part in the face-to-face training and found it to be helpful. Evaluation results regarding the course content showed that the six training participants considered the highly practice-oriented communication training to be helpful for their consultancy work. In contrast, only two participants found the medical-theoretical training content helpful. Of the five students who evaluated the supervision, two felt that it was helpful for their work as *Impf-Guides*.

In a second set of questions, specific activities of the *Impf-Guides* were evaluated. When asked to what extent the* Impf-Guides* had experienced conflicts or challenging situations in the field, four students answered “very rarely” and three answered “never”. Only one student reported having experienced problematic situations “occasionally”. Students were able to describe their experiences in free text format. In summary, the *Impf-Guides* encountered the following challenges during their assignments:

- Disappointed healthcare providers (e.g. pharmacies) regarding access to the vaccine (long, futile wait for vaccine delivery): unwilling to support the mobile vaccination campaign
- Disappointed citizens regarding the effectiveness of the vaccine or citizens who had experienced side effects from vaccination: do not want any further vaccinations
- Anxious citizens regarding possible side effects of the vaccine: refuse vaccination
- General rejection of vaccinations or disinterest in the topic of vaccination
- Language barriers

The *Impf-Guides* primarily used the following arguments in their consultancy work:

- Vaccination campaigns close to home make it easier to get vaccinated
- Protecting oneself and others from severe illness
- Fewer restrictions on participation in social life
- Information on various topics (e.g. vaccine side effects, vaccinations during pregnancy and lactation period, infertility due to vaccinations, how mRNA vaccines work)
- Information about the necessity of booster vaccinations

In the last set of questions, all eight students rated their subjective learning success through their work as *Impf-Guides *as high, particularly with regard to the benefits for their future careers as doctors (*M*=2; *SD*=1.1) and for future communication with patients (*M*=1.5; *SD*=0.5). 

## 4. Discussion and conclusion

The project aimed at increasing vaccination willingness (and thus the COVID-19 vaccination rate) in socially challenged city districts of Munich through information, consulting and a mobile vaccination service. At the time the project was carried out, vaccine hesitancy regarding COVID-19 was a widespread phenomenon worldwide [15], [16]. Vaccine hesitancy is also known from other vaccinations, e.g. against influenza, and is estimated at approximately 8 to 15% in some studies [15]. With regard to COVID-19-related vaccine hesitancy, a review by Rahbeni et al. calculates a pooled rate of approximately 32% in the general population, with significant variations between different countries and population groups [16]. The reasons for vaccine hesitancy are manifold and can be attributed to a lack of trust in science and available vaccines, difficult access to or lack of availability of healthcare facilities, low educational level or support for radical political parties [9], [10], [15], [17]. This makes projects and information campaigns on vaccination all the more important, even though the evidence for the benefits of such projects is still scarce [18]. Studies show that complex (e.g. target group-specific communication, information and knowledge transfer, and improved access to vaccines) and dialogue-oriented interventions (e.g. mobilising advocates for vaccination within defined target groups, using social media or mass media) are most effective [4], [5], [18], [19]. 

It is possible that the dialogue-oriented *Impf-Guides* project, in combination with other components, could have had a greater impact in reducing vaccine hesitancy among the Munich population; however, the project was not planned and implemented in conjunction with other educational measures. The lack of integration of multidisciplinary teams (which, in addition to medical professionals, also include psychologists and/or social workers) to ensure a target group-specific approach can also be seen as a conceptual limitation of the *Impf-Guides. *Furthermore, the target number of 50 *Impf-Guides *could not be achieved, which limited their reach in the city districts. In addition, the use of other communication channels, e.g. in the form of social media, would have been useful. Furthermore, the project was implemented at a time when COVID-19 protective measures were being scaled back throughout Germany, which is likely to have had a negative impact on vaccination willingness [20]. A critical examination of the evaluation results highlights the challenges involved in implementing the project described here, but also confirms the fundamentally sound approach underlying it.

Conducting the training courses for the *Impf-Guides *involved a great deal of organisational effort. While 17 interested students took part in the first training course, only five attended the second. Overall, participants described the practical communication training as particularly helpful for their work as *Impf-Guides*. As the students' interest in the project waned over time, after the second training course only online learning materials were made available on the learning platform. Only eight of a total of 31 *Impf-Guides* completed the questionnaire evaluating their consulting activities in the city districts. All eight *Impf-Guides* rated their personal learning outcomes from the project as high, both for their future medical work and for their communication with patients. However, in light of the low response rate and the lack of prior piloting of the questionnaire instruments, the evaluation results should be interpreted with caution. It is possible that the students had become generally tired of evaluations, as numerous COVID-19-related surveys and studies were conducted at German universities during the pandemic – as evidenced, among other things, by two special editions of the GMS Journal for Medical Education on the topic of “teaching in times of COVID-19” [21], [22]. Furthermore, the subjectivity of the student feedback must be taken into account.

The *Impf-Guides *mainly reached people who were already open to COVID-19 vaccination. The evaluation of vaccinated citizens only provides a non-representative insight into the population of Munich, as only people who were vaccinated were surveyed. The response rate among vaccinated citizens was 18%. The majority of people who were vaccinated had already received a vaccination and therefore presumably already had a good level of health literacy [9], [19]. This assumption is underlined by the free text responses of those vaccinated, who predominantly cited self-protection and protection of others as their reasons for vaccination. Although it is quite likely that people consulted by the *Impf-Guides* also got vaccinated outside of the mobile vaccination campaigns, no figures are available in this regard. It is possible that the intended target groups (especially socioeconomically disadvantaged people and people with a migrant background) were not reached to a sufficient extent. A look at the native language of those vaccinated suggests that this group was not sufficiently reached by the project. There are various reasons for not reaching the target group – for example, medical *Impf-Guides* do not come from the social environment of the target group and were therefore unable to adequately address the problems faced by the target group (i.e., limited access to the health system, lack of health literacy) [19]. In addition, multilingual students were not explicitly recruited as *Impf-Guides.*

Regardless of vaccination campaigns, it is worth considering whether trained and, possibly, multilingual students could act as “health guides” to advise the population on general health, prevention and health promotion issues. The systematic involvement of students as health and prevention advisors can contribute to the acquisition of relevant higher-level skills (see chapter VIII.4 of NKLM 2.0) on the one hand, and to the promotion of health literacy among the population on the other [9], [19]. To achieve these goals, a curricular expansion of learning locations should be established in cooperation with public health care institutions.

## Notes

### Authorship

The authors Jan M. Zottmann and Johanna Huber share the first authorship.

### Authors’ ORCIDs

- Jan M. Zottmann: [0000-0002-3887-1181]
- Johanna Huber: [0009-0005-9518-730X]
- Matthias J. Witti: [0000-0002-5758-1948]
- Martin R. Fischer: [0000-0002-5299-5025]

## Acknowledgements

We would like to thank the project staff of the city of Munich (especially Renate Binder, Christian Breu, Annette Gottstein-Vetter, Annette Gröger, Hubert Schiefer); the Student Council of Human Medicine at LMU Munich (represented by Cedric Smets); all student *Impf-Guides* and especially our project student Hussam Albardawel; our DAM colleagues Sabine Koerth, Sven Sarbu-Rothsching and Marc Weidenbusch; all lecturers of the *Impf-Guides* training courses, in particular the staff of the Aicher Group (represented by Maximilian Hinkofer and Joachim Weidringer) and the AKIM conflict management program of the City of Munich (represented by Sven von Braumüller and Michael Wübbold). 

## Competing interests

The authors declare that they have no competing interests. 

## Supplementary Material

Impf-Guides training programme, course content and lecturers

Survey “COVID-19 vaccination”

Questionnaire for evaluating the “Impf-Guides”

## Figures and Tables

**Table 1:**
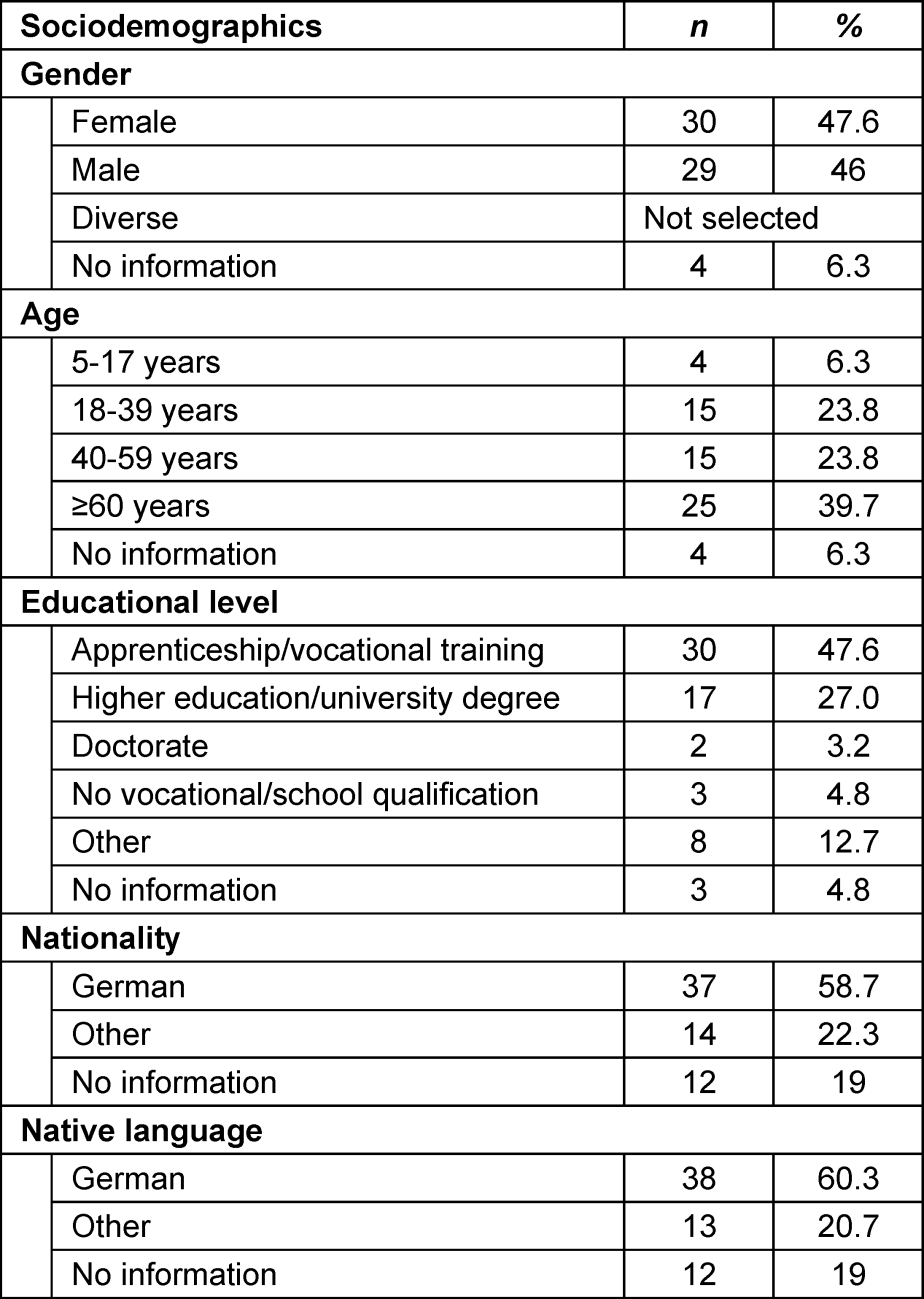
Sociodemographic data of citizens vaccinated through a mobile vaccination campaign in Munich

**Table 2:**
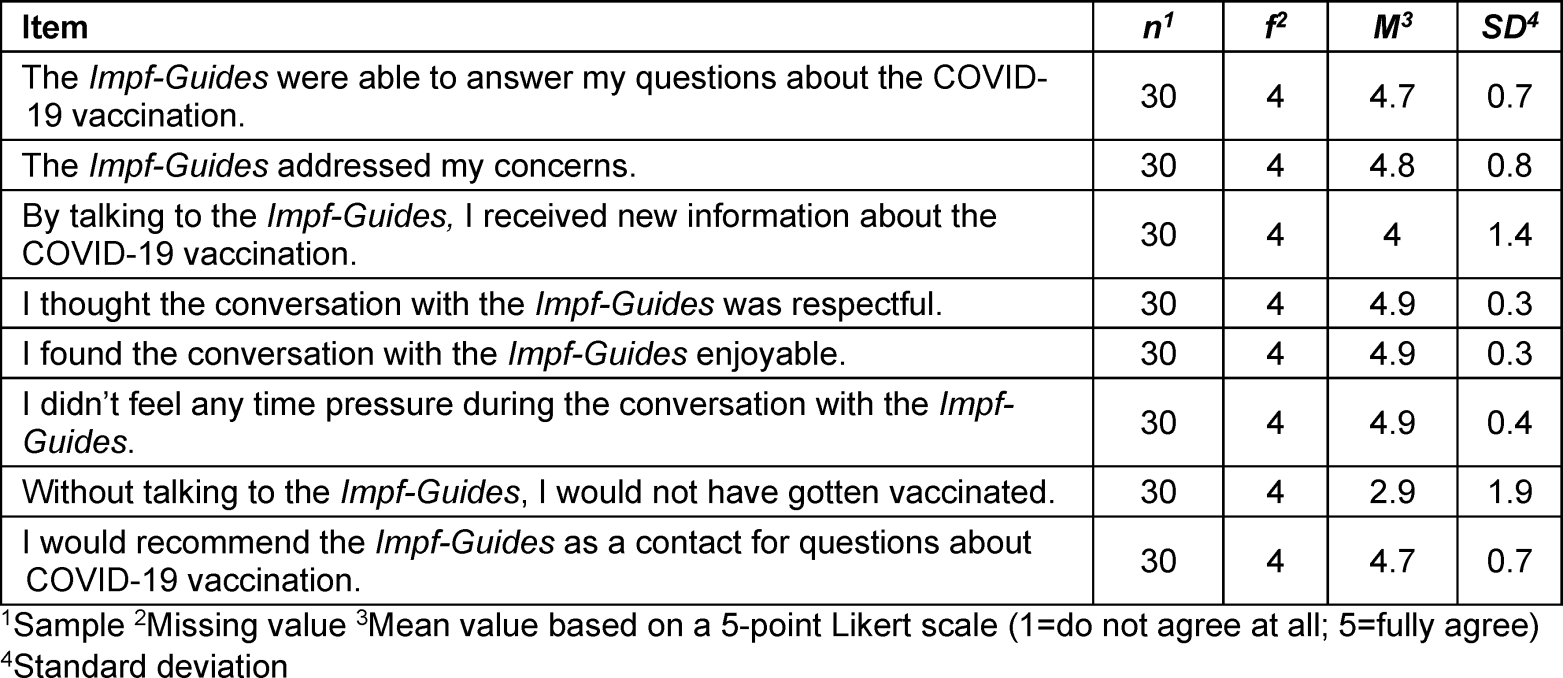
Evaluation of the *Impf-Guides* by vaccinated citizens in Munich (March 2022 to June 2022)

**Table 3:**
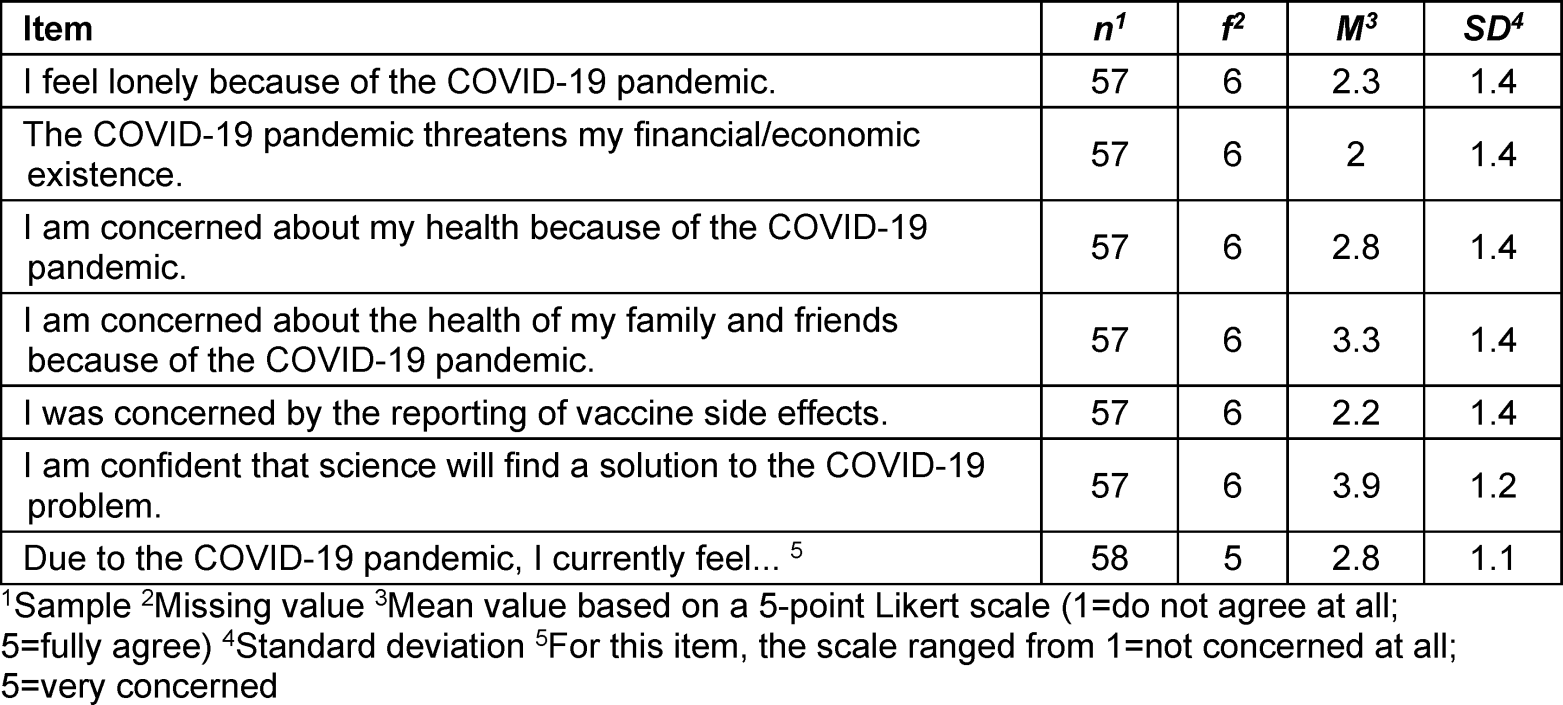
Evaluation of the personal situation during the pandemic by Munich citizens (March 2022 to June 2022)
