# Supplementary material for: Impf-Guides – experiences with a project aimed at increasing vaccination willingness in Munich during the COVID-19 pandemic
Source: GMS J Med Educ. 2026 Apr 15;43(4):Doc45. doi: 10.3205/zma001839 (PMC13124530; doi:10.3205/zma001839)
Supplement: Impf-Guides training programme, course content and lecturers [file JME-43-45-s-001.pdf]

Attachment 1: Impf-Guides training programme, course content and lecturers

Time	Topic	Content	Lecturers
9:30	Welcome		City of Munich<br>Health Department<br>Dean of Studies in Human Medicine at LMU Munich
9:40	Introducing the project	- Project aim: Increase willingness to be vaccinated<br>- Data: Vaccination rates in Munich<br>- Target group of project<br>- Role definition of the *Impf-Guides*:<br>Field consultants and coordination of mobile vaccination campaigns<br>- Structure and organisation of the consulting teams<br>- Exemplary procedure in the city district: Preparation phase (e.g. field exploration), implementation phase in the city district (consulting and advertising for mobile vaccination campaigns and support in carrying out these campaigns)<br>- Materials for the *Impf-Guides*<br>o Backpacks<br>o Multilingual flyers<br>o Letter of authorisation as "ID"<br>o Information folder with important phone numbers and additional multilingual information material	City of Munich<br>Health Department
10:00	City districts	- Definition of city districts (increased infection rates and social challenges, e.g. number of social welfare recipients, proportion of migrants)<br>- Geographical location of the selected city districts<br>- Profiles of the selected city districts	City of Munich<br>Health Department
10:20	Opportunities for conversation in public spaces	- Planning the consultancy assignment<br>- Attitude as an *Impf-Guide*<br>- At the location, e.g. tandem check (do we know what to do?)<br>- During the assignment, e.g. setting and approach<br>- After the assignment, e.g. evaluation of the conversation	AKIM conflict management program of the<br>City of Munich

Attachment 1 to Zottmann JM, Huber J, Witti MJ, Jacobs F, Mahl T, Schmidt A, Fischer MR. *Impf-Guides – experiences with a project aimed at increasing vaccination willingness in Munich during the COVID-19 pandemic*. GMS J Med Educ. 2026;43(4):Doc45. DOI: 10.3205/zma001839

Time	Topic	Content	Lecturers
11:00	Break		
11:15	Coronavirus vaccinations: implementation in Munich	- Concept and locations of vaccination centres<br>- Vaccination teams<br>- Mobile vaccination campaigns<br>- Available vaccines<br>- Vaccination figures per calendar week	Munich vaccination centre, Aicher Group
11:45	Vaccination myths versus vaccination facts	- Reasons for vaccine hesitancy<br>- Conversation techniques that promote vaccination<br>- How do vaccines work?<br>- Evidence-based sources on the topic of vaccination (e.g. BZgA, IQWiG)<br>- Herd immunity<br>- 13 coronavirus vaccination myths fact-checked<br>- Dealing with vaccination myths in conversation<br>- RKI Epidemiologisches Bulletin on vaccination recommendations	Munich vaccination centre, Aicher Group
12:30	Lunch		
13:30	Communication and de-escalation training	- Communication models & examples of practical application<br>o Schulz von Thun<br>o interculturality<br>o B.I.B. model<br>o Iceberg model<br>- Recommended action in conflict-ridden/challenging situations<br>- Exercise in small groups + debriefing<br>- De-escalation & self-defence<br>o Breakwell's phase model<br>o Self-defence § 32 STGB	Anti-aggression trainer as well as members of the Institute of Medical Education (DAM) at LMU Munich
16:30	Course completion & evaluation		Dean of Studies in Human Medicine at LMU Munich

Attachment 1 to Zottmann JM, Huber J, Witti MJ, Jacobs F, Mahl T, Schmidt A, Fischer MR. *Impf-Guides – experiences with a project aimed at increasing vaccination willingness in Munich during the COVID-19 pandemic*. GMS J Med Educ. 2026;43(4):Doc45. DOI: 10.3205/zma001839
