# Supplementary material for: Impf-Guides – experiences with a project aimed at increasing vaccination willingness in Munich during the COVID-19 pandemic
Source: GMS J Med Educ. 2026 Apr 15;43(4):Doc45. doi: 10.3205/zma001839 (PMC13124530; doi:10.3205/zma001839)
Supplement: Survey “COVID-19 vaccination” [file JME-43-45-s-002.pdf]

Attachment 2: Survey “COVID-19 vaccination”

Attachment 2 to Zottmann JM, Huber J, Witti MJ, Jacobs F, Mahl T, Schmidt A, Fischer MR. *Impf-Guides – experiences with a project aimed at increasing vaccination willingness in Munich during the COVID-19 pandemic*. GMS J Med Educ. 2026;43(4):Doc45. DOI: 10.3205/zma001839

evasys	Survey "COVID-19 vaccination"	evasys

Klinikum der LMU München

Institut für Didaktik und Ausbildungsforschung in der Medizin

DAM

Bitte so markieren: ☐ ☒ ☐ ☐ ☐ Bitte verwenden Sie einen Kugelschreiber oder nicht zu starken Filzstift. Dieser Fragebogen wird maschinell erfasst.

Korrektur: ☐ ■ ☐ ☒ ☐ Bitte beachten Sie im Interesse einer optimalen Datenerfassung die links gegebenen Hinweise beim Ausfüllen.

1. Welcome to the survey "COVID-19 vaccination"!

In cooperation with the health department of the City of Munich and the medical students of the Ludwig-Maximilans-Universität München (LMU), we are conducting a project to increase the vaccination rate in Munich.

We, that is scientists at the Institut für Didaktik und Ausbildungsforschung in der Medizin at LMU Klinikum. We would like to ask you about your opinion on the COVID-19 vaccination and how you became aware of the vaccination. The goal of this survey is to improve the information available on COVID-19 vaccination. The survey is anonymous, which means we don't know who you are. Also, participation in the survey is voluntary and you can cancel the survey at any time if you no longer wish to answer the questions. By participating in the survey, you give your consent for your checked answers to be used for research purposes. It is not possible to draw any conclusions about your person. You will need about 10 minutes to complete the survey. If you agree with taking the survey, please check "yes". Please do not forget to click on "Submit" at the end of the survey. If you do not wish to participate in the survey, please check "no".

Thank you for participating in the survey.

Have a great day!

Institute for Medical Education, University Hospital LMU Munich

1.1 I want to participate in the survey on COVID-19 vaccination. ☐ Yes ☐ No

2. Personal details

2.1 How old are you? Please enter your age in years.

[ ][ ]

2.2 What is your gender? ☐ Female ☐ Male ☐ Diverse ☐ Prefer not to answer

2.3 What is your nationality?

☐ Bosnia and Herzegovina ☐ Germany ☐ France
☐ Great Britain ☐ Kosovo ☐ Croatia
☐ Montenegro ☐ Austria ☐ Russia
☐ Suisse ☐ Serbia ☐ Turkey
☐ Ukraine ☐ Other:

2.4 Other:

[ ]

2.5 What is your native language? Please choose only one language.

☐ Bosnisch ☐ German ☐ English
☐ French ☐ Croatian ☐ Montenegrin
☐ Russian ☐ Serbian ☐ Turkish
☐ Ukrainian ☐ Other:

2.6 Other:

[ ]

MUSTER

MUSTER

2. Personal details [Fortsetzung]

2.7 What is your highest educational qualification? Please select a degree from the list.

- ☐ Apprenticeship/ Professional training
- ☐ Graduate/ University degree
- ☐ Promotion
- ☐ No professional/ school education
- ☐ Other:

2.8 Other:

2.9 Are you currently employed?

- ☐ Yes, full time
- ☐ Yes, part time
- ☐ Yes, occasionally (e.g. Minijob)
- ☐ No, still in education
- ☐ No, retired/ pensioned
- ☐ No, not at all

2.10 How long have you lived in Munich?

- ☐ A few months
- ☐ 1 to 5 years
- ☐ 5 to 10 years
- ☐ More than 10 years

2.11 Which corona vaccination did you receive today?

- ☐ 1. Vaccination
- ☐ 2. Vaccination
- ☐ 3. Vaccination
- ☐ 4. Vaccination

2.12 Why didn't you let yourself be vaccinated so far?

With how many stars would you rate the following statement?
1 star = You do not agree at all.
5 stars = You fully agree.

2.13 The vaccination offer in my district made the decision to vaccinate easy.

Do not agree at all ☐ ☐ ☐ ☐ ☐ Fully agree

3. Questions on COVID-19 vaccination

3.1 How did you learn about the COVID-19 vaccination campaign in your district? You can choose more than one answer.

- ☐ Information brochure/Direct mail from the City of Munich
- ☐ Talk with a vaccination guide (In Munich, medical students from the LMU inform about the COVID-19 vaccination)
- ☐ Neighbours
- ☐ Familiy/Friends
- ☐ Medical doctor
- ☐ Agentur für Arbeit
- ☐ Münchner Tafel
- ☐ Kindertagesstätte
- ☐ Kindergarten
- ☐ School
- ☐ Other:

MUSTER

MUSTER

3. Questions on COVID-19 vaccination [Fortsetzung]

3.2 Other:

3.3 Did you have a conversation with an "Impf-Guide"? (In Munich, medical students from the LMU inform about the COVID-19 vaccination) ☐ Yes ☐ No

With how many stars would you rate the following statements?
1 star = You do not agree at all.
5 stars = You fully agree.

3.4	The "Impf-Guides" were able to answer my questions about the COVID-19 vaccination.	Do not agree at all ☐	☐	☐	☐	☐ Fully agree
3.5	The "Impf-Guides" addressed my concerns.	Do not agree at all ☐	☐	☐	☐	☐ Fully agree
3.6	By talking to the "Impf-Guides", I received new information about the COVID-19 vaccination.	Do not agree at all ☐	☐	☐	☐	☐ Fully agree
3.7	I thought the conversation was respectful.	Do not agree at all ☐	☐	☐	☐	☐ Fully agree
3.8	I found the conversation with the "Impf-Guides" enjoyable.	Do not agree at all ☐	☐	☐	☐	☐ Fully agree
3.9	I didn't feel any time pressure during the conversation.	Do not agree at all ☐	☐	☐	☐	☐ Fully agree
3.10	Without talking to the "Impf-Guides", I would not have gotten vaccinated.	Do not agree at all ☐	☐	☐	☐	☐ Fully agree
3.11	I would recommend the "Impf-Guides" as a contact for questions about COVID-19 vaccination.	Do not agree at all ☐	☐	☐	☐	☐ Fully agree

3.12 Why did you decide to get vaccinated?

3.13 I have already been through a COVID-19 infection. ☐ Yes ☐ No

4. How do you assess your situation since the COVID-19 pandemic?

With how many stars would you rate the following statements?
1 star = You do not agree at all.
5 stars = You fully agree.

4.1	I feel lonely because of the COVID-19 pandemic.	Do not agree at all ☐	☐	☐	☐	☐ Fully agree
4.2	The COVID-19 pandemic threatens my financial/ economic existence.	Do not agree at all ☐	☐	☐	☐	☐ Fully agree

MUSTER

MUSTER

4. How do you assess your situation since the COVID-19 pandemic? [Fortsetzung]

4.3	I am concerned about my health because of the COVID-19 pandemic.	Do not agree at all ☐	☐	☐	☐	☐	Fully agree
4.4	I am concerned about the health of my family and friends because of the COVID-19 pandemic.	Do not agree at all ☐	☐	☐	☐	☐	Fully agree

With how many stars would you rate the following statement?
1 star = Far too light
5 stars = Far too strict

4.5	The current COVID-19 measures are:	Far too light ☐	☐	☐	☐	☐	Far too strict

4.6 Which sources/media do you mainly use to find out about COVID-19? You can select more than one answer.

☐ Television ☐ Radio ☐ Social media and messenger services e.g. Twitter, Facebook
☐ Scientific publications ☐ Newspapers and magazines ☐ Other:

4.7 Other:

With how many stars would you rate the following statements?
1 star = You do not agree at all.
5 stars = You fully agree.

4.8	I was concerned by the reporting of vaccine side effects.	Do not agree at all ☐	☐	☐	☐	☐	Fully agree
4.9	I am confident that science will find a solution to the COVID-19 problem.	Do not agree at all ☐	☐	☐	☐	☐	Fully agree

With how many stars would you rate the following statement?
1 star = I feel not concerned at all.
5 stars = I feel very concerned

4.10	Due to the COVID-19 pandemic, I currently feel:	Not concerned at all ☐	☐	☐	☐	☐	Very concerned

MUSTER
