# Supplementary material for: Impf-Guides – experiences with a project aimed at increasing vaccination willingness in Munich during the COVID-19 pandemic
Source: GMS J Med Educ. 2026 Apr 15;43(4):Doc45. doi: 10.3205/zma001839 (PMC13124530; doi:10.3205/zma001839)
Supplement: Questionnaire for evaluating the “Impf-Guides” [file JME-43-45-s-003.pdf]

Attachment 3: Questionnaire for evaluating the “Impf-Guides”

Attachment 3 to Zottmann JM, Huber J, Witti MJ, Jacobs F, Mahl T, Schmidt A, Fischer MR. *Impf-Guides – experiences with a project aimed at increasing vaccination willingness in Munich during the COVID-19 pandemic*. GMS J Med Educ. 2026;43(4):Doc45. DOI: 10.3205/zma001839

MUSTER

Institut für Didaktik und Ausbildungsforschung in der Medizin

Kontakt: johanna.huber@med.uni-muenchen.de

DAM

Bitte so markieren: ☐ ☒ ☐ ☐ ☐ Bitte verwenden Sie einen Kugelschreiber oder nicht zu starken Filzstift. Dieser Fragebogen wird maschinell erfasst.

Korrektur: ☐ ■ ☐ ☒ ☐ Bitte beachten Sie im Interesse einer optimalen Datenerfassung die links gegebenen Hinweise beim Ausfüllen.

1. Welcome to the evaluation of the project "Impf-Guides"!

Dear students,

Thank you for participating in the "Impf-Guides" project.

We would like to know how you feel about working as an "Impf-Guide":
- What aspects of the project do you think are working well?
- Where do you think there is room for improvement in the project?
- How do you personally benefit from this project?

Your feedback will help us to further develop the “Impf-Guides” project so that future vaccination campaigns can be carried out as effectively and target-group-specifically as possible.

Please allow approximately 20 to 30 minutes to complete the survey. As some of the questions are open-ended, it will take a little longer to answer than standard teaching evaluations.

Your information is anonymous and voluntary. You can cancel your participation at any time.

We look forward to your feedback!

Thank you very much for your participation.

Best regards,
Jan Zottmann and Johanna Huber
Institute of Medical Education

2. Training for "Impf-Guides" and supervision

2.1 Did you participate in the "Impf-Guides" training on February 26 or April 2?
☐ Yes ☐ No

2.2 Overall, did you find the training content helpful for your work as an "Impf-Guide"?
☐ Yes ☐ Partially ☐ No ☐ I do not know

*The training covered information about the *city districts*, the *role of "Impf-Guides"*, the topic of *“vaccination myths and facts”*, how *to initiate conversations in the city districts*, and *communication and conflict training*.

2.3 What did you feel was lacking? Is there any additional content you would have liked to see?

2.4 Did you find the medical training content helpful for your work as a vaccination guide?
☐ Yes ☐ Partially ☐ No ☐ I do not know

MUSTER

MUSTER

2. Training for "Impf-Guides" and supervision [Fortsetzung]

2.5 What did you feel was lacking? Is there any additional content you would have liked to see?

2.6 Did you find the communication training helpful for your work as an "Impf-Guide"?
- ☐ Yes
- ☐ Partially
- ☐ No
- ☐ I do not know

2.7 What did you feel was lacking? What kind of content would you have liked to see?

2.8 Did you attend either of the two supervision sessions that were offered?
- ☐ Yes
- ☐ No
- ☐ I did not know that supervision existed.

2.9 Did you find the supervision helpful for your work as an "Impf-Guide"?
- ☐ Yes
- ☐ Partially
- ☐ No
- ☐ I do not know

2.10 Why did you find supervision helpful?

2.11 What did you feel was lacking in the supervision? Is there anything else you would have liked to see?

3. Working as an Impf-Guide

3.1 I worked as an "Impf-Guide":
- ☐ on the street.
- ☐ in the office.
- ☐ both on the street and in the office.

3.2 How often have you encountered challenging situations while working as an "Impf-Guide" on the street?
- ☐ Never
- ☐ Very rare
- ☐ Occassionally
- ☐ Often
- ☐ Very often

3.3 Please describe one or two situations that you found particularly challenging.

MUSTER

MUSTER

3. Working as an Impf-Guide [Fortsetzung]

3.4 How did you manage to resolve or cope with the situation(s) described above?

3.5 How many consultations do you estimate you have conducted?

3.6 What percentage of people do you think you were able to convince to get vaccinated?

3.7 Which arguments did you use to convince people to get vaccinated? Please describe them.

3.8 What arguments did the people you approached on the street use to reject the advice about vaccination given by the "Impf-Guides"? Please summarise these arguments in bullet points.

3.9 Overall, would you consider the "Impf-Guides" project to be a success? ☐ Yes ☐ No ☐ I do not know

3.10 Why do you think the "Impf-Guides" project is successful?

3.11 Why do you think the "Impf-Guides" project is not successful?

4. Teamwork

4.1	The collaboration within the "Impf-Guides" team was:	Excellent ☐	☐	☐	☐	☐ Poor	☐ K.A.
4.2	The working atmosphere within the "Impf-Guides" team was:	Very pleasant ☐	☐	☐	☐	☐ Not pleasant at all	☐ K.A.

MUSTER

4. Teamwork [Fortsetzung]

4.3	My tandem partners were able to support me in difficult communication situations.	Completely applies ☐	☐	☐	☐	☐ Do not apply at all	☐ N.A.

5. Personal development

5.1	My work as an "Impf-Guide" will benefit me in my future career as a doctor.	Completely applies ☐	☐	☐	☐	☐ Do not apply at all	☐ N.A.
5.2	My work as an "Impf-Guide" will be helpful for my future communication with patients.	Completely applies ☐	☐	☐	☐	☐ Do not apply at all	☐ N.A.
5.3	My work as an "Impf-Guide" will be particularly helpful for me in future communication with patients with a migrant background.	Completely applies ☐	☐	☐	☐	☐ Do not apply at all	☐ N.A.
5.4	My work as an "Impf-Guide" is beneficial for my personal development.	Completely applies ☐	☐	☐	☐	☐ Do not apply at all	☐ N.A.

6. Overall rating of the "Impf-Guides" project

6.1 Which aspects of working as an "Impf-Guide" did you particularly enjoy?

6.2 What improvements do you see for the “Impf-Guides” project?

6.3 Would you consider working as an "Impf-Guide" again in future? ☐ Yes ☐ No ☐ I do not know

6.4 What are your reasons for not wanting to work as an "Impf-Guide" again?

6.5 What would you need to be able to work as an "Impf-Guide" again in the future?

MUSTER
